# Onset of summer monsoon in Northeast India is preceded by enhanced transpiration

**DOI:** 10.1038/s41598-019-55186-8

**Published:** 2019-12-09

**Authors:** Rohit Pradhan, Nimisha Singh, Raghavendra P. Singh

**Affiliations:** Land Hydrology Division, Space Applications Centre (ISRO), Ahmedabad, 380 015 India

**Keywords:** Hydrology, Hydrology

## Abstract

Variations in isotopic composition of water vapor in the atmosphere is an important indicator of the processes within the hydrological cycle. Isotopic signature of water vapor and precipitation can be helpful in partitioning evaporation and transpiration fluxes. It is well known that transpiration from forested regions supplies a significant amount of vapor to the atmosphere in monsoon and post-monsoon seasons. Here, we utilize observations from Tropospheric Emission Spectrometer (TES), Atmospheric Infra-Red Sounder (AIRS) and simulation models to ascertain that transpiration is dominant in the forests of Northeast India (NE) during pre-monsoon season. Our results show an increase in δD of 78.0 ± 7.1‰ and in specific humidity of 3.1 ± 0.2 g kg^−1^ during the pre-monsoon months of April-May compared to January-February. In the monsoon months of July-August, δD reduces by 53.0 ± 6.5‰ albeit the specific humidity increases by 3.4 ± 0.2 g kg^−1^. Using joint observations of specific humidity and isotope ratio in lower troposphere, we discern the moisture sources over NE India in pre-monsoon and monsoon seasons and posit the role of transpiration in continental recycling during pre-monsoon season.

## Introduction

Indian subcontinent receives maximum rainfall from June to September^1^, which accounts for major source of annual precipitation. Northeast (NE) India receives highest rainfall during the Indian Summer Monsoon as compared to other parts of Indian subcontinent^2^. Forests of NE India cover 64% geographical area of the region^3^ and are regarded as the northern most limit of tropical rainforests in the world^4^. This region is a major biodiversity hotspot of India and is home to a wide variety of plant species^5^. Previous studies have considered NE India as a distinct macro-region within the Indian landmass^6^. This region is dominated by dynamic weather that is influenced by water vapor in the atmosphere arising from oceanic as well as terrestrial sources like vegetation, open water bodies etc. Orography of the region also plays a major role in causing precipitation^2^. Evapotranspiration (ET) is an important component of hydrological cycle that plays a vital role in surface hydrology, vegetation dynamics and energy balance at the terrestrial surface. Continental recycling of ET in NE India is well understood for the post monsoon period^7^ but not for the pre-monsoon and transition periods. Goroshi *et al*.^8^ found a strong positive correlation between ET and insolation in NE India implying that insolation and ambient air temperature are major stress factors affecting ET in this region. Correct quantification of evaporation and transpiration in any hydrological model is necessary to reduce uncertainties in the hydrological budget. In this regard, studying the isotopic signature of water (H_2_^16^O and HDO) can provide evidence about the dominant prevailing process amongst evaporation and transpiration^9,10^.

Rainforests form a crucial component of global carbon and hydrological cycles^11^. Wright *et al*.^12^ reported that Amazon rainforests play an important role in destabilizing the atmosphere resulting in early onset of wet season. Forests of NE India are vulnerable to changing climate conditions^5^. Monsoon precipitation over the region has been on a continuous decline since 1950s^13^. Breitenbach *et al*.^14^ studied the stable isotopes in precipitation during 2007–08 in Meghalaya, NE India and proposed that there is a shift from northwestern continental moisture source (during winter) to southern marine source (during Indian summer monsoon). Western Disturbance (WD) influences precipitation during winter months with moisture originating from Mediterranean Sea and mid-west Atlantic Ocean^15^. This phenomenon is more pronounced in Western and Central Himalayas as compared to Eastern Himalayan region^16^. During summer months, when most of India is dry, NE India receives a significant amount of precipitation. Nor’westers typically occur in summer months and are characterized by thunderstorms that develop due to intensive atmospheric vortices and convective processes^17^. Intensive localized evaporation from open water bodies like rivers and lakes provides moisture for generation of these thunderstorms, which typically cover distances of less than 50 km^18^. However, large scale moistening of the lower troposphere during pre-monsoon months driven by transpiration from forests has not been well studied. Precipitation based studies conducted earlier^18^ only provide Nor’westers as possible explanation of pre-monsoon precipitation.

Conventional models and techniques for discharge estimation have helped constrain river discharge fluxes to the ocean^19^. However, lack of well-distributed measurements and difficulty of modeling the latent heat fluxes usually result in poor constraints on evaporation and transpiration components at catchment scale. Measurement of stable isotope ratios of oxygen and hydrogen is used to decouple evaporation and transpiration^9,20^. Evaporation causes the resulting vapor to be depleted in heavier isotopes whereas transpiration at steady state does not cause fractionation^9,21^. Analysis of stable water isotopes in rivers and lake water across the globe have been used to partition various fluxes within the hydrological cycle^10,22^. Isotopic analysis of precipitation has been widely carried out to understand moisture sources^18,23–26^. Using observations of δD and δ^18^O in precipitation and comparing them with Global Meteoric Water Line (GMWL) provides information about source and transport of moisture over a region^27,28^.

Satellite-based remote sensing of water isotopes in atmosphere is an effective tool to understand certain hydrological processes^29–32^. Satellite sensors like SCanning Imaging Absorption spectroMeter for Atmospheric CHartographY (SCIAMACHY) onboard ENVISAT-1 and Tropospheric Emission Spectrometer (TES) onboard Aura have provided valuable long-term records of isotopic ratio in atmospheric water vapor^33,34^. Satellites can measure concentrations of HDO and H_2_^16^O in atmosphere, but limitations in sensitivity of current instruments do not allow the retrieval of H_2_^18^O. As a result, observations of D/H ratio are only available from space-based platforms. However, with joint measurements of isotopic composition of water vapor and specific humidity, it is possible to retrace the history of condensation and mixing processes associated with an air parcel^35^. Retrieval of HDO from SCIAMACHY uses the shortwave infrared channels and provides total columnar concentration^34^, whereas TES retrieval uses the thermal infrared channels to provide vertical profile of HDO^33^. Isotopic composition of water vapor differs with that of precipitation due to different sources and hence, remote sensing gives new information about the hydrological cycle, which is unattainable from surface. We carried our study with two major objectives: (a) to determine the source of moisture in lower troposphere over NE India during pre-monsoon months and (b) to study the role of this atmospheric moistening in convection process resulting in pre-monsoon precipitation.

## Study Area and Data Used

**Figure 1:**
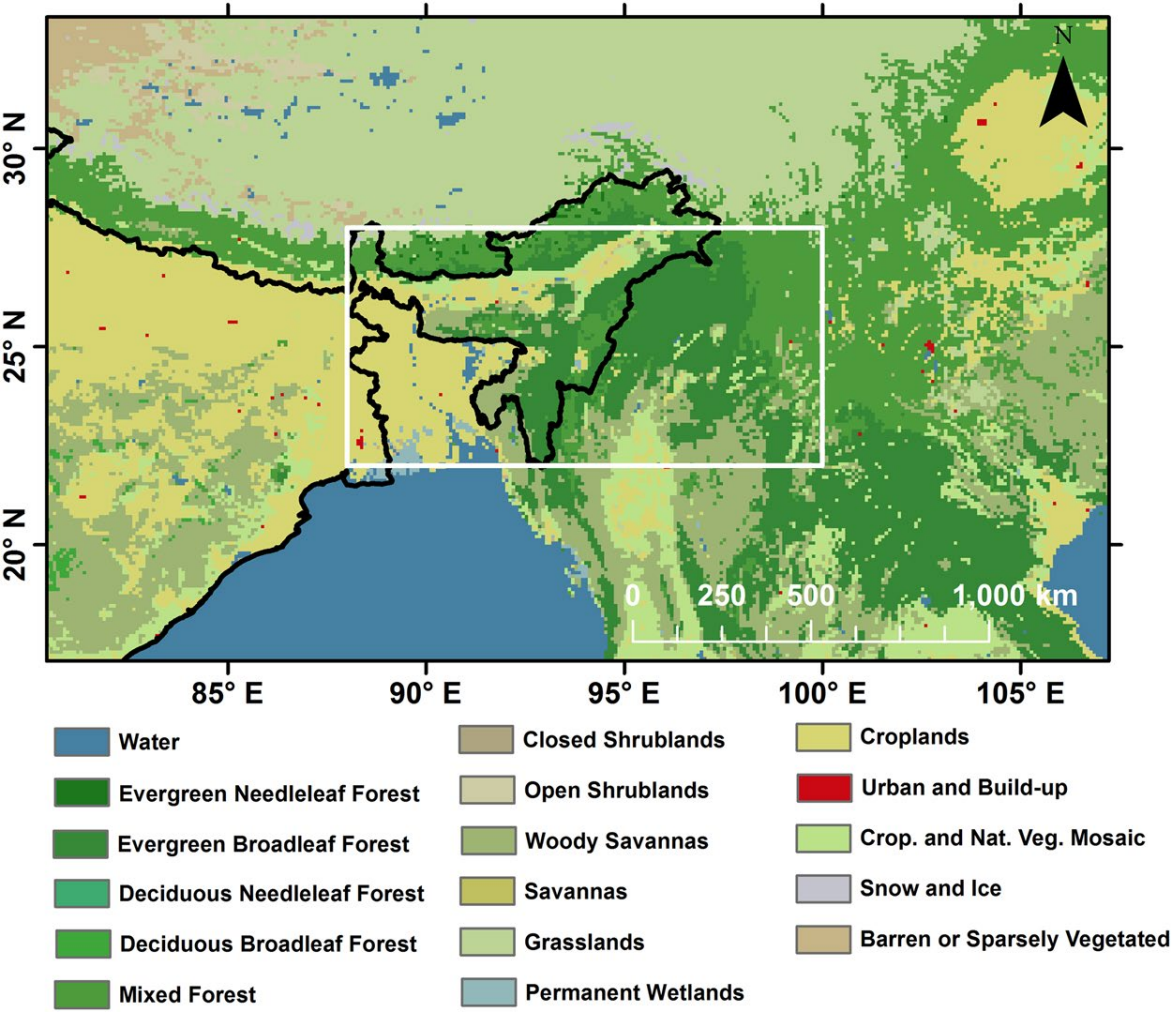
MODIS Land Use/Land Cover^36^ for 2009 (White box represents the study area).

Study was carried over NE Indian region with bounding box 22°–28° N and 88°–100° E. Figure 1 shows the study area overlaid on MODIS Land Use/Land Cover^36^ (LULC) for 2009. The size of bounding box was chosen to accommodate atleast two adjoining passes of Aura satellite. Different forest types cover major area of this region, along with the flood plains of Brahmaputra in Assam and Bangladesh. This region receives majority of rainfall during the Indian Summer Monsoon with normal onset date between 1–10 June every year^37^. The climate of this region is sub-tropical and it receives some of the highest rainfall in the world. Mawsynram in Meghalaya, India is the wettest place on Earth and lies within the study area.

We have used TES Level 2 (Nadir-Lite-Version 6) retrievals of HDO and H_2_O profiles^38^ for the period 2006–2009 during which the data points were most dense. TES measures HDO and H_2_O concentrations in the water vapor at 17 pressure levels during ascending (~1345 LT at equator) and descending (~0145 LT) passes of Aura satellite with footprint of 5.3 km × 8.4 km^33^. Individual TES observations have large biases as reported in earlier literature^33,39,40^. Worden *et al*.^33^ noted that TES data in its initial phase showed bias of ~5% in estimation of HDO, which accounted for ~50‰ δD bias for values near zero and smaller bias error for values much below zero. However, with subsequent revisions of TES data, these biases have been accounted for and the same have been incorporated in the provided Nadir Lite v6 product used in our present study^39,41^. Errors such as smoothing errors (given by averaging kernel), systematic and noise errors etc. are inherent to the algorithm for retrieving trace gases from space-based spectrometric techniques^42^. TES retrieval algorithm performs joint retrievals of multiple species including HDO and H_2_O to reduce these errors^33^ in the measurement and a full quantification of individual errors is provided in the product file. Although individual data points may be biased, averaging data points over a larger grid and increasing the number of independent observations reduces the uncertainty. Point observations by TES have a precision of ~10–15‰ which reduces to 1–2‰ when averaging over larger regions^31,43,44^.

**Figure 2:**
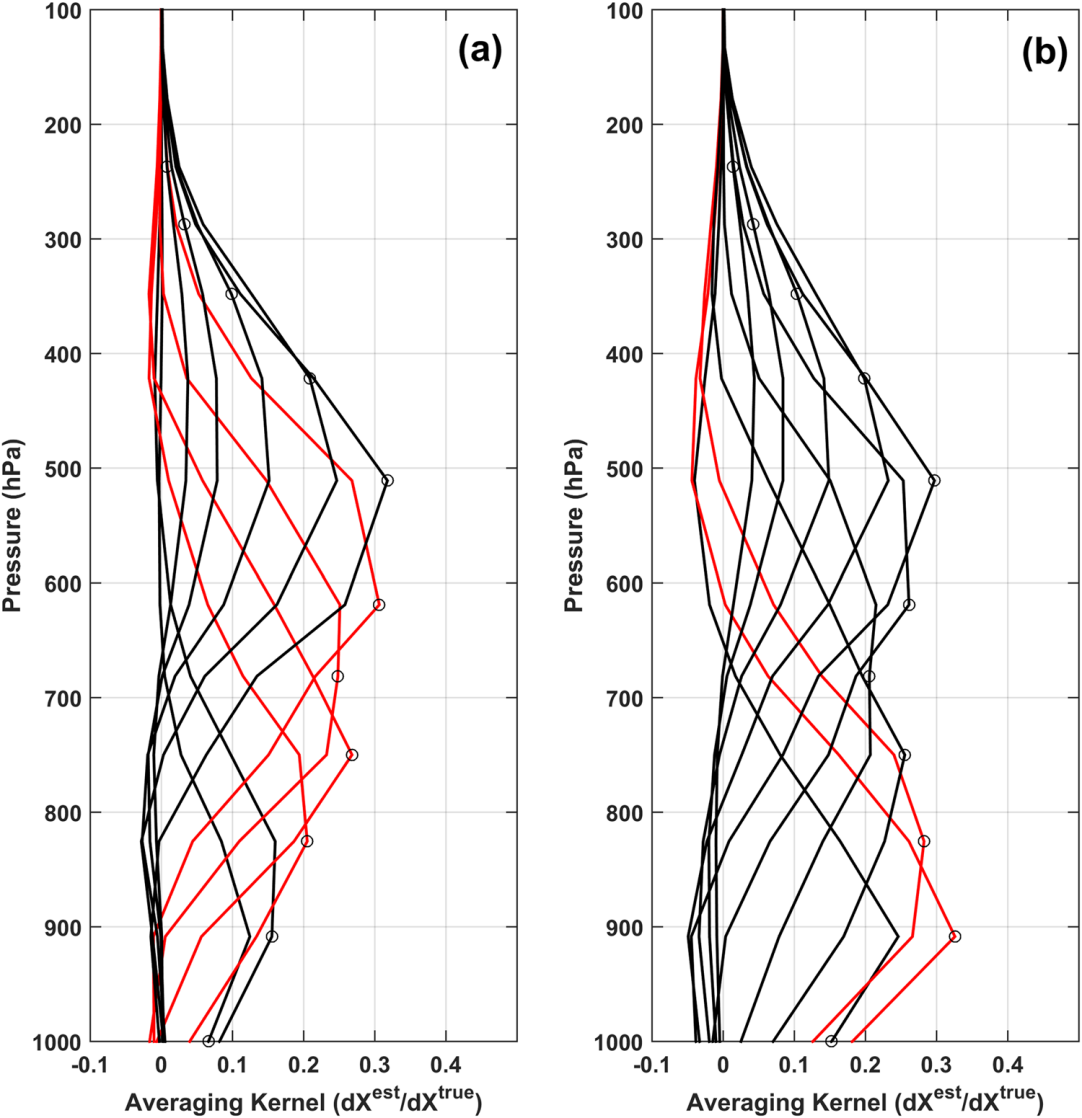
Mean averaging kernel for TES measurements of HDO for Apr-May 2006 over (**a**) NE India and (**b**) Bay of Bengal.

Data was filtered using quality control criteria described by Worden *et al*.^45^ where average cloud optical depth was <0.4 (clear-sky retrievals) and degree of freedom of signal >1. These criteria allow only cloud-free observations to be considered, as cloud cover reduces the vertical resolution of TES observation^39^. Providing these control filters on TES dataset affects the number of valid points but does not influence the qualitative nature of results obtained^12^. Averaging kernel represents the sensitivity of log(R) at a given pressure level to that of ratios at other pressure levels, where R is the ratio of concentration of HDO to H_2_O. Figure 2 shows the mean averaging kernel of valid TES retrievals over NE India and Bay of Bengal for Apr-May 2006. TES retrievals over NE India are sensitive in the 825–619 hPa range with reduced sensitivity close to surface. This is expected as TES observations are known to be sensitive in lower to mid-troposphere^39^. Observations over Bay of Bengal (bounding box 8°–18° N and 85°–92° E) show better sensitivity in the 908–825 hPa range (Fig. 2b) and this is suitable to derive the isotopic signature of an evaporative source originating from here.

Atmospheric InfraRed Sounder (AIRS) and Advanced Microwave Sounding Unit (AMSU) based TqJoint product of specific humidity (q) and temperature (T) was used in the study^46^. Level 3 AIRX3STD v6 daily retrievals of q and T at 1° × 1° at standard pressure levels between 1000–300 hPa was obtained for 2006–2009. Co-located observations of AIRS and TES datasets was possible because they lie within the A-train of satellites and take observations few minutes apart. European Center for Medium-Range Weather Forecasting Interim Reanalysis (ERA-Interim) modelled monthly wind velocity at 850 hPa was used to determine general wind direction during different months^47^.

MODIS 16-day Enhanced Vegetation Index^48^ (EVI) at 0.05 degrees was obtained for the period 2006–2009. Data from Global Network of Isotopes in Precipitation^49^ (GNIP) provided by International Atomic Energy Agency (IAEA) and isotopic data for rainwater samples collected by Breitenbach *et al*.^14^ at Cherrapunji were used to determine the isotopic signature of precipitation in the study area. Rainfall data was obtained from Global Satellite Mapping of Precipitation^50^ (GSMaP) provided by JAXA at 0.1° × 0.1° resolution and converted to daily rainfall. Daily latent heat flux data was obtained from NCEP/NCAR reanalysis dataset provided by NOAA/ESRL.

## Methodology

### Calculation of VMR and δD profiles

Stable isotope ratio of HDO/H_2_O is usually defined in parts per thousand (per mil- ‰) relative to a standard (Vienna Standard Mean Ocean Water, VSMOW) and given by delta (δ- notation) expressed by the formula^51^,

1 $${\rm{\delta }}{\rm{D}}=[\frac{{({{\rm{HDO}}/{\rm{H}}}_{2}{\rm{O}})}_{{\rm{sample}}}}{{({{\rm{HDO}}/{\rm{H}}}_{2}{\rm{O}})}_{{\rm{VSMOW}}}}-1]\times 1000({\rm{\textperthousand }})$$

where the value of (HDO/H_2_O)_VSMOW_ is 3.11 × 10^−4^. Individual TES retrievals with average cloud optical depth <0.4 and degree of freedom of signal >1 were used for analysis. All valid TES retrievals within the study area were binned over 5-day periods to generate pentad-averaged vertical profiles of water vapor volume mixing ratio (VMR) and δD. TES derived δD at different pressure levels were converted to a single pressure-weighted value using Eq. (2)^52^:

2 $${{\rm{\delta }}{\rm{D}}}_{{\rm{av}}}=\frac{{\sum }_{{\rm{i}}=1}^{4}{{\rm{\delta }}{\rm{D}}}_{{\rm{i}}}{{\rm{q}}}_{{\rm{i}}}{\Delta {\rm{P}}}_{{\rm{i}}}}{{\sum }_{{\rm{i}}=1}^{4}{{\rm{q}}}_{{\rm{i}}}{\Delta {\rm{P}}}_{{\rm{i}}}}$$

where δD_av_ is the pressure-weighted average of 825–619 hPa, δD_i_ is the δD of i^th^ pressure level, q_i_ is TES derived water vapor volume mixing ratio of i^th^ layer and ΔP_i_ is the pressure thickness of i^th^ layer. i = 1, 2, 3, 4 corresponds to 825, 750, 681 and 619 hPa layers of TES retrievals.

AIRS retrieved specific humidity between 850–600 hPa and ERA-Interim modelled wind velocity at 850 hPa were analyzed for the months of February to July during the study period. Weighted mean of daily specific humidity at three pressure levels between 850–600 hPa was computed and averaged over different months. Quiver plots of ERA-Interim monthly wind velocity was overlaid on specific humidity maps.

### Mixing models and q-δ plots

To get an idea about the isotopic signature of soil moisture, data from GNIP and isotopic data for rainwater samples collected by Breitenbach *et al*.^14^ at Cherrapunji were used. Isotopic data of precipitation from two stations (viz. Shillong and Guwahati) within the study region was available at monthly time-steps from the GNIP database along with data points collected by Breitenbach *et al*.^14^ at Cherrapunji during 2007–08. Monthly data from GNIP with number of rain samples >1 were considered. Similarly, we estimate the isotopic composition of an evaporative source from Bay of Bengal by computing the pressure-weighted average of TES observations from 908 to 825 hPa over the region.

When two vapor parcels mix, the resulting parcel’s mixing ratio is the weighted average of their individual mixing ratios. Isotopic composition of the mixed parcel is given by^53^:

3 $${\delta }_{mix}={q}_{2}({\delta }_{2}-{\delta }_{1})\frac{1}{{q}_{mix}}+{\delta }_{1}$$

where ${\delta }_{mix}$ and ${q}_{mix}$ are the isotopic ratio and specific humidity of the resulting mixed air parcel. ${\delta }_{1}\,$is isotopic ratio of free troposphere set between −250‰ to −400‰ at 50‰ steps. ${\delta }_{2}\,$and ${q}_{2}$ represent the isotopic ratio and specific humidity of rising evaporative source, respectively. Subsequently, theoretical curves for pseudo-adiabatic Rayleigh model is given by^12,35^

4 $${\delta }_{Rayleigh}=(\alpha -1)\mathrm{ln}\,(\frac{q}{{q}_{0}})+{\delta }_{0}$$

where ${\delta }_{0}$and ${q}_{0}$ denote isotopic ratio and specific humidity of the initial vapor parcel, ${\delta }_{Rayleigh}$ is the isotopic ratio of resulting vapor after all condensate is removed and *α* is the temperature dependent fractionation factor. Similarly, curves for reversible moist adiabatic process (with precipitation efficiency zero) is represented as^35^

5 $${\delta }_{moa}=({\alpha }_{e}-1)[\frac{q-{q}_{0}}{q-{\alpha }_{e}(q-{q}_{0})}]+{\delta }_{0}$$

where ${\delta }_{moa}$ is the isotopic ratio of resulting vapor when formed condensate is not removed and *α*_*e*_ is the equilibrium fractionation factor. For plotting Rayleigh curves, *α* is set as *α*_*e*_. The initial point for simulating ideal Rayleigh and moist adiabatic processes have been taken at 80% of the specific humidity of the evaporative source. These curves have been modelled for free troposphere with δD = −250‰ only.

Mean monthly specific humidity (AIRS) and δD (TES) for all co-located observations within the study area for period of January-August between 825–619 hPa were used to generate q-δ plots. Seasonal means for January-February, March to May and June to August were also plotted with 1σ variability of each distribution. Individual q-δ data points for these seasons were plotted along with seasonal means and slope of q- δ distribution was computed using a simple linear fit.

### Equivalent potential temperature and atmospheric instability

We studied the AIRS TqJoint specific humidity and temperature profiles to determine the equivalent potential temperature (θ_e_) given by Eq. (6)^54^.

6 $${{\rm{\theta }}}_{{\rm{e}}}={{\rm{\theta }}}_{{\rm{d}}}\exp \,(\frac{{{\rm{L}}}_{{\rm{V}}}{\rm{q}}}{{{\rm{c}}}_{{\rm{p}}}{\rm{T}}})$$

where θ_d_ is the dry potential temperature, L_v_ is latent heat of vaporization at 273 K, q is water vapor mass mixing ratio, c_p_ is specific heat of dry air at constant temperature and T is air temperature. We computed the area mean of specific humidity and temperature at each pressure level for daytime observations to estimate θ_e_.

Equivalent potential temperature is conserved during changes in pressure of an air parcel (associated with vertical mixing). If θ_e_ reduces with altitude, the atmosphere becomes unstable and vertical mixing occurs. We can quantify this conditional instability as difference between equivalent potential temperature at 850 and 500 hPa pressure levels^12^.

7 $${\rm{Conditional}}\,{\rm{Instability}}\,({\rm{in}}\,{\rm{K}})={{\rm{\theta }}}_{{\rm{e}}}(850\,{\rm{hPa}})-{{\rm{\theta }}}_{{\rm{e}}}(500\,{\rm{hPa}})$$

Large positive value of this temperature difference implies a greater potential for moist convection to occur.

### EVI, ET and precipitation

MODIS 16-day composite EVI at 0.05-degree resolution was analyzed for mid-March and mid-May and averaged across four years. Area averaged mean of EVI was computed for all four years to show its temporal variation at 16-day intervals. Daily rainfall data from GSMaP was binned over 5-day periods and area average was computed for each pentad. NCEP/NCAR Reanalysis daily latent heat flux data was converted to pentad-averaged ET by dividing it with the latent heat of vaporization of pure water at 20 °C.

## Results and Discussion

### Variations in water vapor and δD

**Figure 3:**
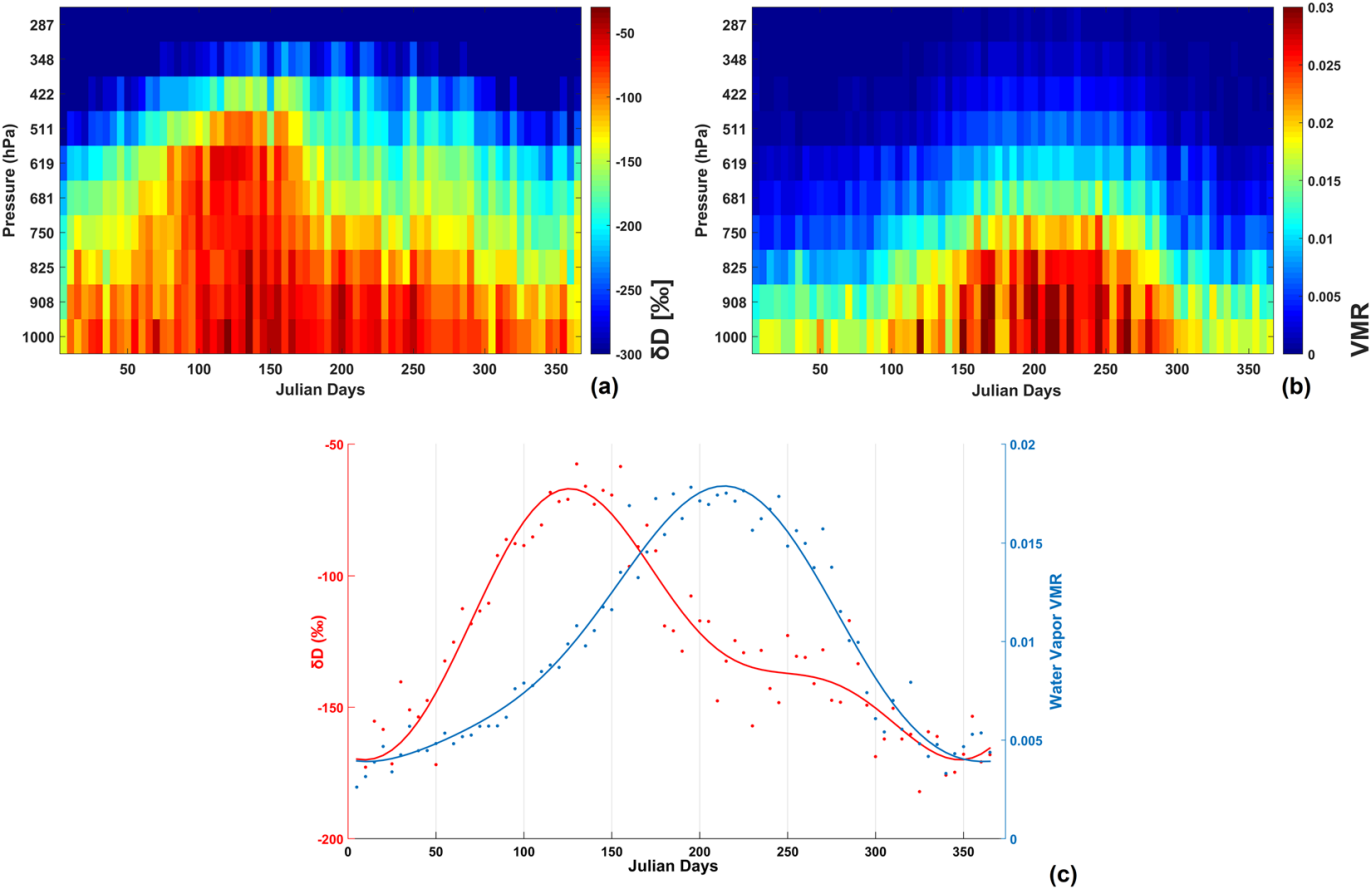
TES retrieved (**a**) δD and (**b**) water vapor VMR at ten pressure levels averaged over 5-day periods during 2006–09. (**c**) Line plots of pressure weighted δD and water vapor VMR between 825–619 hPa.

Figure 3a shows TES retrieved inter-annual mean δD values at 10 pressure levels above the surface with x-axis representing time, where each cell along x-direction represents 5-day averaging periods. Usual onset period of monsoon in NE India is around 1–10 June every year^37^ (corresponding to 151–160 Julian Day). However, during the study period, the onset dates were 27–28 May in 2006^37^, 8–10 June in 2007^55^, 31 May-9 June in 2008^56^ and 25 May – 28 June in 2009^57^. It is evident that there is an increase in δD values in pre-monsoon period near the surface and same signal propagates in the mixing layer between 750–619 hPa. Water vapor volume mixing ratio (Fig. 3b,c) follows a nominal increasing trend associated with onset of monsoon in June, even though increase in δD was observed much before that period. Figure 3c shows line plots of pressure-weighted δD and water vapor volume mixing ratio between 825–619 hPa. These two profiles provide an initial hint about some prevailing process during the summer months of April and May. TES is most sensitive between 825–619 hPa and enrichment of vapor at these pressure levels points to mixing of vapor in free troposphere with another isotope rich source.

**Figure 4:**
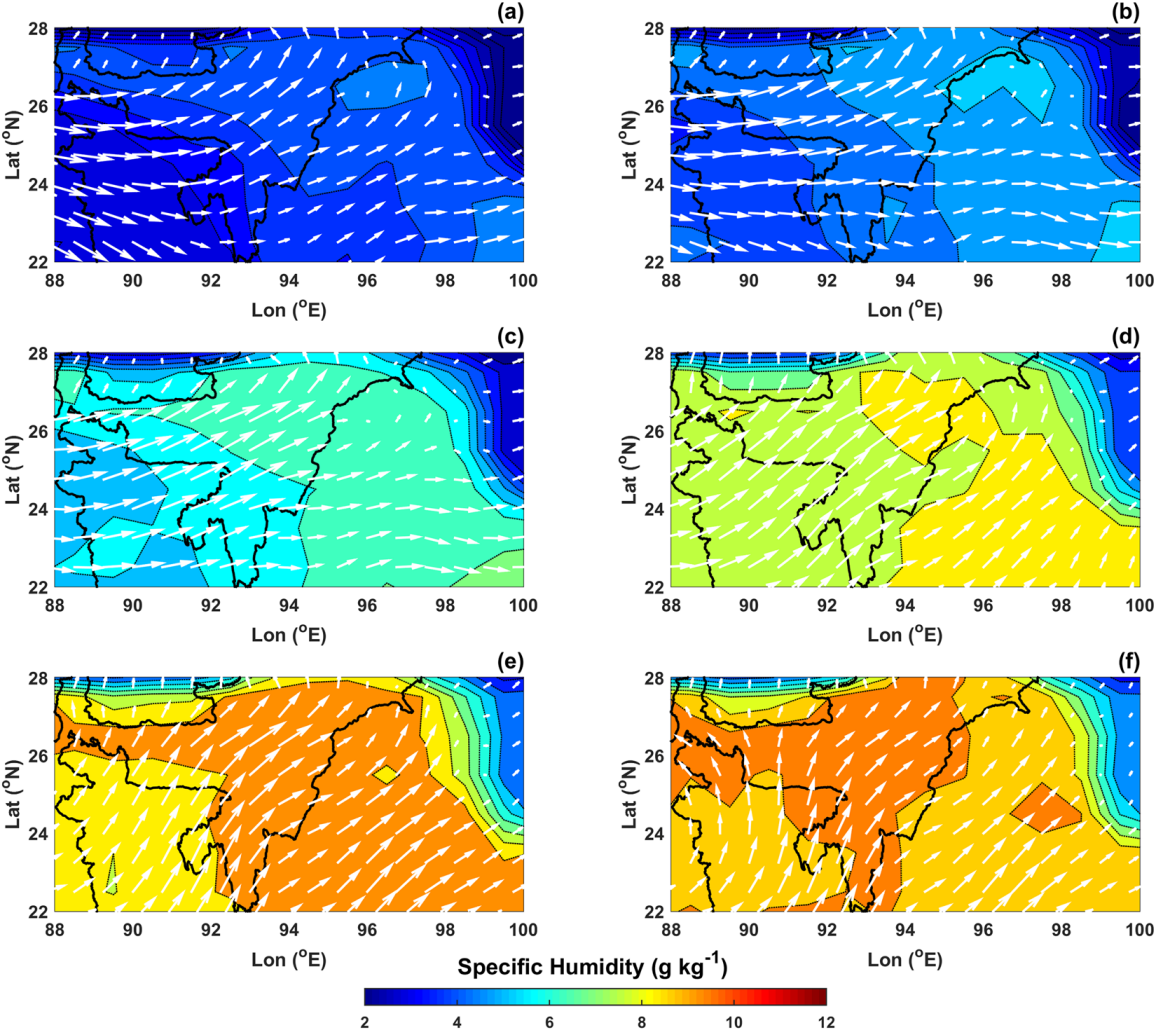
AIRS retrieved specific humidity and ERA-Interim wind velocity (quivers) at 850 hPa for NE India averaged over 2006–09 (**a**-February, **b**-March, **c**-April, **d**-May, **e**-June and **f**-July).

To understand whether this signal is due to local mixing or due to transport of isotope rich water vapor from other regions, AIRS retrieved specific humidity between 850–600 hPa and ERA-Interim modelled wind velocity at 850 hPa were analyzed for the months of February to July. Figure 4 shows the mean monthly specific humidity overlaid with ERA-Interim wind velocity at 850 hPa. During winter months, Western Disturbance carries moisture from Mediterranean Sea and Atlantic Ocean and results in precipitation in Western and Central Himalayas. This phenomenon is less pronounced over NE India and the vapor is mostly dry and depleted during Jan-Feb. During the pre-monsoon period, winds arise from western direction and since most of Indian landmass is dry, increase in δD cannot be attributed to transport of moisture from these regions. Mean specific humidity increased from 3.7 ± 0.1 g kg^−1^ in Jan-Feb to 6.8 ± 0.2 g kg^−1^ in Apr-May, which hints towards local sources of water vapor. The source of this enrichment can also be understood by studying the latitudinal and longitudinal gradients of δD.

**Figure 5:**
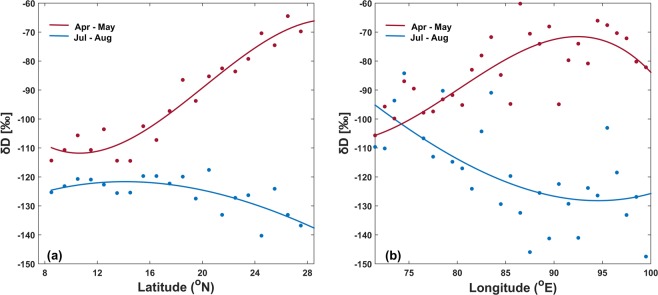
(**a**) Latitudinal (8°N–28°N) and (**b**) longitudinal (72°E–100°E) gradients of weighted δD during Apr-May and Jul-Aug. Width and height of bounding box is same as study area for latitudinal and longitudinal gradients, respectively.

Figure 5a shows latitudinal gradient (South-to-North) of δD between 8°N–28°N for the months of Apr-May and Jul-Aug (East-West bound same as study area: 88°E–100°E). Here, we can clearly observe enrichment of vapor (by ~40‰) in Apr-May as we move northward. Vapor over Bay of Bengal is depleted and cannot be the moisture source for our study area. During monsoon, depletion of vapor is observed as we move northward. Precipitation results in preferential removal of HDO from vapor (arising from Bay of Bengal and carried northward) and depletes the remaining air.

Figure 5b shows longitudinal gradient (West-to-East) of δD between 72°E–100°E for the months of Apr-May and Jul-Aug (North-South bound same as study area: 22°N–28°N). Again, we observe enrichment of vapor (by ~30‰) in Apr-May as we move from western and central India towards NE India. If these regions were the source of vapor for NE India during Apr-May, we would have observed somewhat similar δD. These gradients, along with increase in specific humidity observed during April and May, hint towards local sources of water vapor. During the months of June and July, wind direction changes and monsoonal winds bring more water vapor from Bay of Bengal (south of the region) resulting in further increase in specific humidity.

### Sources of water vapor

**Figure 6:**
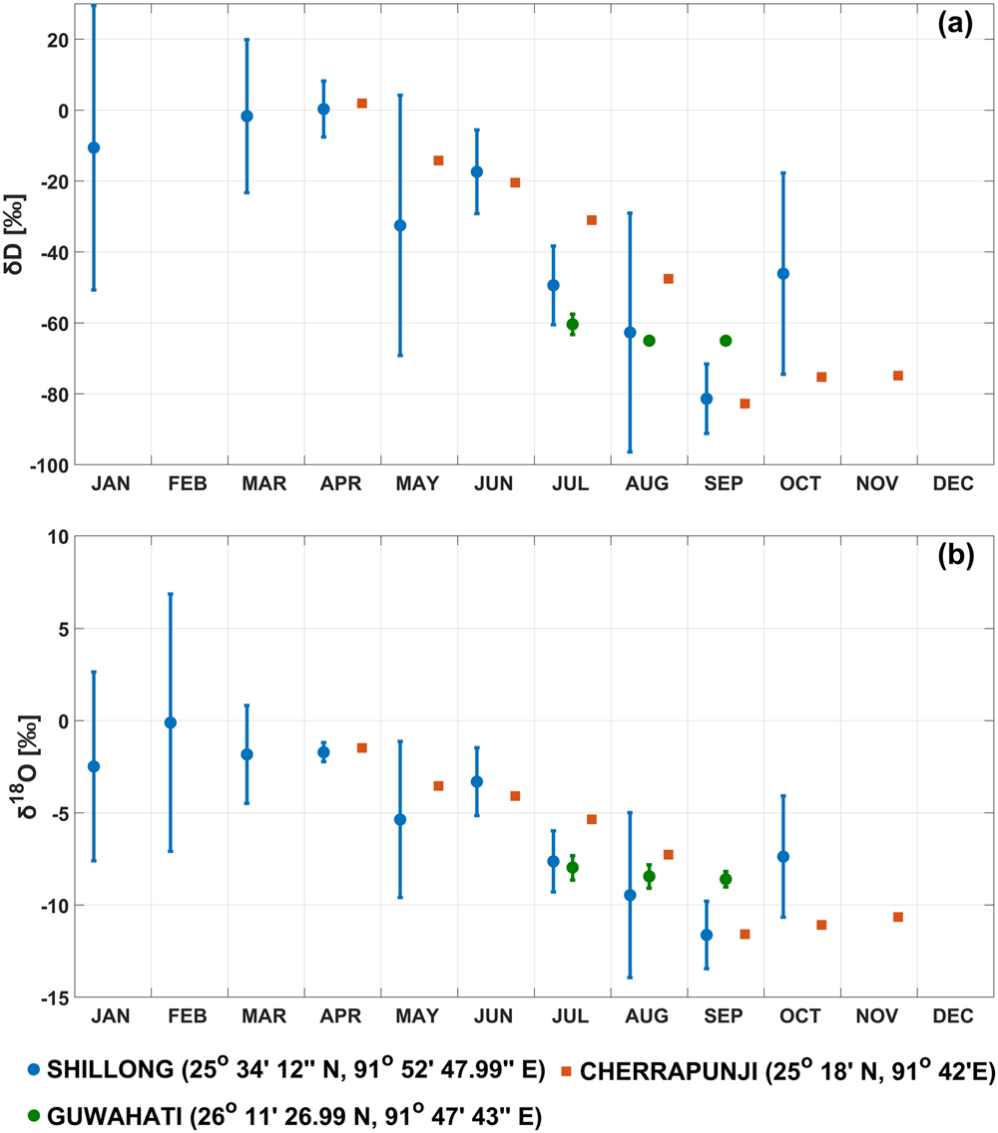
Observed (**a**) δD and (**b**) δ^18^O in precipitation over two stations (Shillong, Guwahati) obtained from GNIP and rainwater samples over Cherrapunji^14^. Data period: Shillong (1966–78), Guwahati (2003–04) and Cherrapunji (2007–08).

Joint measurements of q and δD can help us ascertain whether increase in lower tropospheric humidity during pre-monsoon period happens due to local transpiration or advection from Bay of Bengal or sources from western and central India. Oceanic source of vapor is depleted in heavy isotopes due to fractionation occurring during phase change^58^. However, plant transpiration at steady state does not cause fractionation and the isotopic signature of soil moisture at root zone is similar to that of the transpired vapor^59,60^. Data obtained from GNIP and Breitenbach *et al*.^14^ during April and May (Fig. 6) show that precipitation was more enriched in heavy isotopes (δD ~0 to −20‰; δ^18^O ~ −2 to −5‰). Shillong data showed the highest uncertainty in δ values which were recorded during 1966–1978. Observations at Cherrapunji coincided with our study period and provided the best available estimates. Using these observations, we can assume transpired vapor to have δD ~ −20‰.

**Figure 7:**
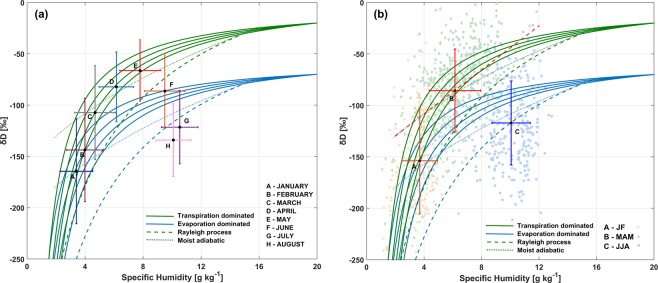
(**a**) *q-δ* diagrams for NE India during the period January-August using co-located AIRS and TES observations. Error bars represent one standard deviation of all observations. (**b**) Seasonal *q-δ* observations for Jan-Feb, Mar-May and Jun-Aug with scatter representing individual observations. Dashed red line represents linear fit of *q-δ* for Mar-May.

TES observations in 908–825 hPa levels showed that vapor arising from Bay of Bengal was relatively depleted (δD~−70‰) during Apr-May. Therefore, the two vapor sources (i.e. transpired and ocean-evaporate sources) are isotopically distinct. When vertical mixing of these two sources occurs with the free troposphere, the isotope ratio of final vapor will be different. To understand this, we plot the theoretical mixing curves of these two sources of water vapor with the free troposphere^12,35,53^. Mixing curves (solid lines in Fig. 7) with varying δ_1_ (i.e. isotope ratio of free troposphere ranging from −250‰ to −400‰ at 50‰ steps) and δ_2_ (i.e. isotope ratio of source; −20‰ for transpiration source and −70‰ for ocean evaporate source) were plotted. Initial specific humidity values of the two end-members were assumed to be q_1_ = 1.5 g kg^−1^ and q_2_ = 20 g kg^−1^. These are close approximations to conditions in NE India during the pre-monsoon season. When two vapor sources mix, there is a possibility of condensation and subsequent precipitation leading to further fractionation and more depleted vapor. Dashed lines in Fig. 7 represent the depleted vapor due to Rayleigh process (precipitation efficiency = 1) and dotted lines represent the reversible moist adiabatic process (precipitation efficiency = 0). These are highly idealized theoretical mixing curves and may not exactly represent the real world scenarios. However, they can be helpful in interpretation of the actual observations.

Figure 7a shows the mean monthly specific humidity (AIRS) vs δD (TES) for all co-located observations within the study area for period of January-August between 825–619 hPa. During the months of April-May, we observe an increase in mean specific humidity (3.1 ± 0.2 g kg^−1^) and δD (78.0 ± 7.1‰) compared to January-February. Error terms represent the standard error in estimation of mean whereas error bars in Fig. 7 represent the standard deviation of monthly observations. There is a gradual increase in specific humidity from winter months to pre-monsoon season. However, a sharp increase in δD was observed in March compared to previous months, which increased further in April and May. Figure 7b shows the seasonal means and scatter of individual co-located q-δ observations. If the moistening above boundary layer (825–600 hPa) is dominated by upward mixing of local transpiration, then higher q should be associated with higher δD values during pre-monsoon season. Instead, if this moistening is dominated by transport from evaporative/oceanic sources, then higher q should be associated with lower δD values. We clearly observe a positive slope of linear fit between q and δD for pre-monsoon months with greater number of observations showing high q and high δD values. We observe a shift from dry and depleted vapor in winter months to moist and enriched vapor in pre-monsoon months. Since transpiration source can explain these observations, this q-δ diagram presents evidence that transpiration is indeed the dominant process during pre-monsoon period in the region. With onset of monsoon in June, depleted vapor arrives from Bay of Bengal and mixes with the overlying atmosphere resulting in net decline of δD but increase in specific humidity. In July and August, when monsoon is in full swing, there is drastic reduction in δD as more evaporate arrives from the sea.

### Analysis of meteorological and vegetation parameters

**Figure 8:**
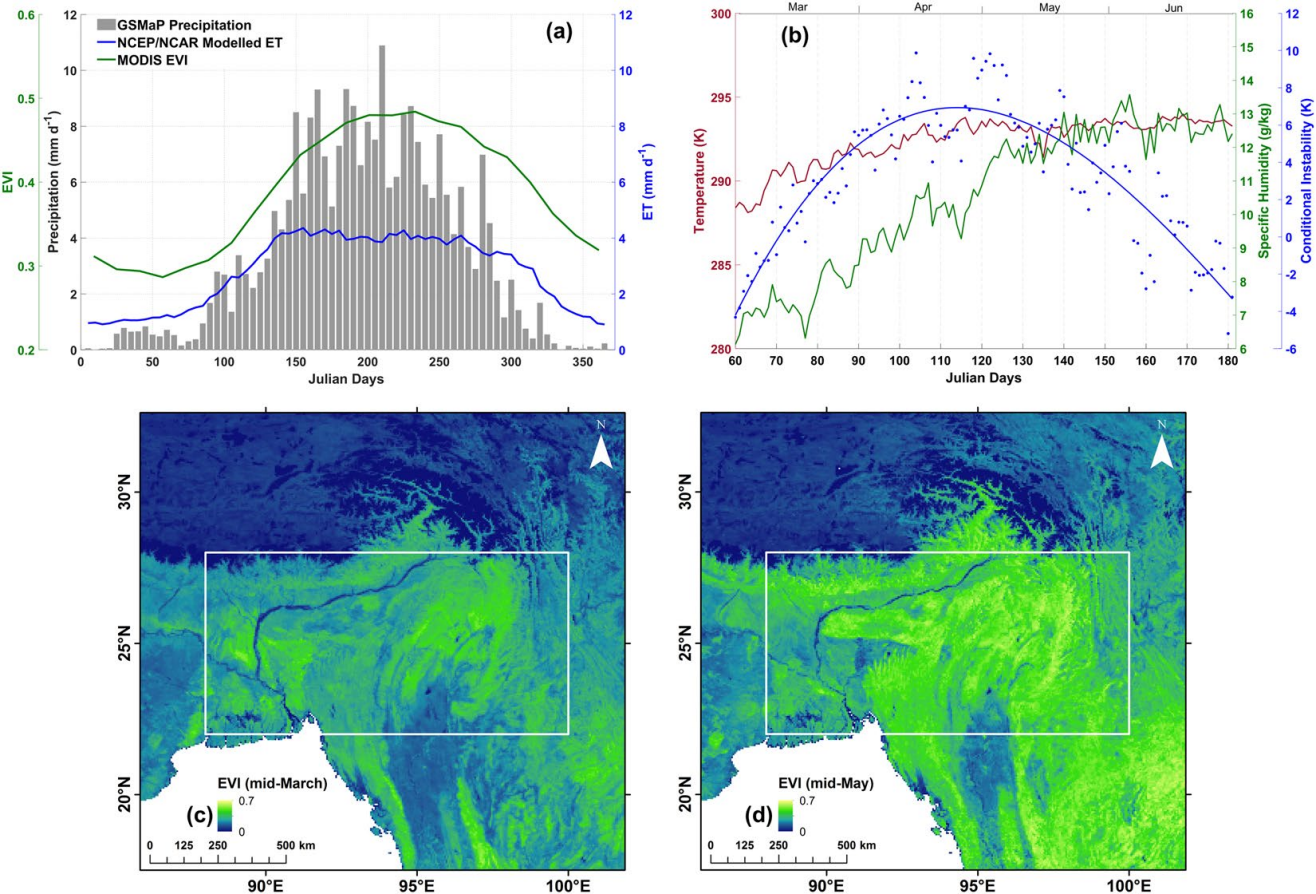
(**a**) Temporal variability of ET from NCEP/NCAR Reanalysis, precipitation from GSMaP and MODIS EVI over study region averaged for the period 2006–09. (**b**) Daytime conditional instability parameter, temperature and specific humidity at 850 hPa at daily time step for March to June. (**c**) EVI during mid-March and (**d**) EVI during mid-May averaged across the four years.

Figure 8a shows the pentad-averaged GSMaP precipitation and NCEP/NCAR Reanalysis ET over the study region. Substantial amount of pre-monsoon precipitation occurs in the months of April-May. During summer months, Nor’westers are known to cause thunderstorms in late afternoon. The scale of activity of Nor’westers is small (<50 km) compared to our study area and form due to intense convection. Due to the difference in spatial scale of these processes and the different observational periods, it is safe to assume that the effect of Nor’westers on TES observations would be minimal. However, the contribution of Nor’westers is important in regards to the supply of moisture to forested regions during pre-monsoon season. Intense rainfall caused by Nor’westers helps redistribute water from open bodies like rivers, lakes etc. to vegetated or forested regions in NE India, thereby increasing soil-moisture and plant-available water supply, which in turn results in enhanced transpiration.

TES and AIRS observations show a large scale moistening of the lower troposphere dominated by transpiration. The role of this increased vapor on atmospheric stability can be understood by studying the equivalent potential temperature at different pressure levels. Negative vertical gradient of equivalent potential temperature in atmosphere results in conditional instability that can trigger convection process. In other words, high positive values of conditional instability parameter increases the chance of moist convection. Figure 8b shows the area averaged daily conditional instability parameter for March to June along with specific humidity and temperature at 850 hPa. Close to boundary layer, the temperature change from March to May is small whereas specific humidity almost doubles. The conditional instability parameter is negative in the first few weeks but starts increasing by the end of March. The atmosphere is most unstable during April to mid-May with high positive values of the instability parameter (~4–10 K). Figure 8c,d show the MODIS 16-day composite EVI for mid-March and mid-May, respectively. From these figures, it is evident that NE Indian region undergoes greening during the pre-monsoon months leading to intense transpiration. This coincides with the time when atmosphere is unstable to convection resulting in increased chance of precipitation. The process of moist convection also causes the enriched transpired vapor to rise higher in altitude and mix with the free troposphere. Transpiration being the dominant source of vapor during this time must contribute substantially to this precipitation, resulting in continental recycling of ET.

## Conclusion

In this study, we used multiple satellite observations of various meteorological and isotopic parameters to determine the source of vapor in free troposphere above NE India and ascertained increase in local transpiration during pre-monsoon months. TES observations indicated sharp increase in δD values during April and May between 825–619 hPa pressure levels. Mean δD values increased from −153.9 ± 6.0‰ in Jan-Feb to −75.8 ± 3.8‰ in Apr-May whereas mean specific humidity increased from 3.7 ± 0.1 g kg^−1^ in Jan-Feb to 6.8 ± 0.2 g kg^−1^ in Apr-May. q-δ plots of co-located TES-AIRS observations point to a transpiration dominated vapor source mixing with the lower troposphere. Pre-monsoon atmospheric moistening could explain the large-scale atmospheric instability over the region resulting in increased chance of convection. The technique of interpreting q-δ diagrams could be implemented in other parts of India to understand the evolution and transport of water vapor in the atmosphere. Further study is required to determine the role of this enhanced transpiration in atmospheric feedback mechanisms and its response to declining monsoon precipitation in the region.

## Data Availability

All data is available online and free of charge. TES and AIRS profiles, MODIS Land cover and EVI data is available at NASA’s EARTHDATA portal https://earthdata.nasa.gov. NCEP Reanalysis data is provided by the NOAA/OAR/ESRL PSD, Boulder, Colorado, USA from their website at https://www.esrl.noaa.gov/psd/. The ECMWF ERA-Interim reanalysis data is available at the European Centre for Medium Range Weather Forecast website https://www.ecmwf.int/. GNIP dataset is available at IAEA WISER portal https://nucleus.iaea.org/wiser. GSMaP precipitation data is available from JAXA’s EORC portal https://sharaku.eorc.jaxa.jp/GSMaP/.
